# Peroxiredoxin I and II as novel therapeutic molecular targets in cervical cancer treatment through regulation of endoplasmic reticulum stress induced by bleomycin

**DOI:** 10.1038/s41420-024-02039-7

**Published:** 2024-05-31

**Authors:** Hu-Nan Sun, Da-Yu Ma, Xiao-Yu Guo, Ying-Ying Hao, Mei-Hua Jin, Ying-Hao Han, Xun Jin, Taeho Kwon

**Affiliations:** 1 Stem Cell and Regenerative Biology Laboratory, College of Life Science & Biotechnology, Heilongjiang Bayi Agricultural University, Heilongjiang, Daqing 163319 China; 2 Department of Biochemistry and Molecular Biology, Tianjin Medical University Cancer Institute and Hospital, National Clinical Research Center for Cancer, Tianjin’s Clinical Research Center for Cancer, Key Laboratory of Cancer Prevention and Therapy, Tianjin, 300060 China; 3 Primate Resources Center, Korea Research Institute of Bioscience and Biotechnology (KRIBB), Jeongeup-si, Jeonbuk 56216 Republic of Korea; 4 Department of Applied Biological Engineering, KRIBB School of Biotechnology, Korea National University of Science and Technology (UST), Daejeon, 34113 Republic of Korea

**Keywords:** Cancer, Cell biology

## Abstract

Cervical cancer, significantly affecting women worldwide, often involves treatment with bleomycin, an anticancer agent targeting breast, ovarian, and cervical cancers by generating reactive oxygen species (ROS) to induce cancer cell death. The Peroxiredoxin (PRDX) family, particularly PRDX1 and 2, plays a vital role in maintaining cellular balance by scavenging ROS, thus mitigating the damaging effects of bleomycin-induced mitochondrial and cellular oxidative stress. This process reduces endoplasmic reticulum (ER) stress and prevents cell apoptosis. However, reducing PRDX1 and 2 levels reverses their protective effect, increasing apoptosis. This research highlights the importance of PRDX1 and 2 in cervical cancer treatments with bleomycin, showing their potential to enhance treatment efficacy by managing ROS and ER stress and suggesting a therapeutic strategy for improving outcomes in cervical cancer treatment.

## Introduction

Based on authoritative data analysis, Cervical cancer is the fourth leading cause of death from malignant tumors in women worldwide [1]. Annually, there are ~500,000 new cases and over 230,000 deaths among females due to this disease [2]. Cervical cancer disproportionately affects women in developing countries, accounting for ~85% of global cases [3]. Currently, the treatment methods for cervical cancer include surgery, radiotherapy, and chemotherapy [4]. Prominent chemotherapy drugs for advanced cervical cancer encompass cisplatin, 5-fluorouracil, and bleomycin [5–7]. Bleomycin (BLM), a significant anticancer drug, is extensively employed in treating various malignancies, such as breast cancer, ovarian cancer, and cervical cancer [7–9]. Notably, bleomycin is known to cause pulmonary fibrosis as a side effect [10]. Distinguished from other anti-tumor drugs, BLM impedes the protein synthesis in cancer cells, thus inhibiting their growth and spread [11]. In this study, we explore the mechanism by which bleomycin regulates apoptosis in human cervical cancer SiHa cells, focusing on its role in inducing cancer cell death.

Reactive oxygen species (ROS) constitute a highly reactive group of oxidants, including entities like superoxide anions, hydrogen peroxide, and hydroxyl radicals [12]. Predominantly generated by the mitochondria within cells [13], ROS are integral to cellular metabolism. However, their overabundance can inflict substantial cellular damage, potentially culminating in cell death [14, 15]. There is a close relationship between ROS and endoplasmic reticulum (ER) stress, excessive ROS can directly or indirectly trigger ER stress [16], a critical cellular response. The ER, a crucial cellular organelle, plays a key role in protein synthesis, folding, and modification [17, 18]. It oversees over one-third of the cell’s protein synthesis and folding processes, and additionally, it manages lipid biosynthesis and calcium homeostasis, thereby playing an essential role in preserving cellular stability [19, 20]. Various factors, including genetic mutations and external stimuli, can impede accurate protein folding and translation, which leads to a buildup of incorrectly folded or unfolded proteins inside the ER. The buildup of substances in the ER causes ER stress, triggering the unfolded protein response (UPR) [19]. The UPR functions to reestablish ER homeostasis by curtailing protein synthesis, enhancing protein folding and transport mechanisms, and stimulating ER-associated degradation systems. In situations of prolonged or severe ER stress that exceeds the ER’s ability to recover, the UPR triggers apoptosis to eliminate compromised cells [21, 22]. Glucose-regulated protein 78 (GRP78), a member of the heat shock protein 70 family and commonly found in association with the ER membrane, plays a vital role in this process. In normal conditions, GRP78 remains inactive, binding to key downstream signaling pathways like activating transcription factor 6 (ATF6), protein kinase RNA-like ER kinase (PERK), and inositol-requiring enzyme 1 (IRE1). An abundance of improperly folded proteins in the ER causes GRP78 to detach from these pathways, thereby activating it. This activation initiates the UPR, which inhibits protein synthesis and enhances proper protein folding [23]. While primarily aimed at regulating cellular homeostasis, sustained or severe ER stress activates pro-apoptotic factors through the UPR, driving the cell toward apoptosis [24]. ER stress is closely associated with the development of a range of diseases, such as cancer, diabetes, and cardiovascular disorders [25–27]. The growing body of research underscores the significant connection between ROS and ER stress. This study seeks to elucidate the dynamics between ROS and ER stress, exploring how ROS regulation impacts ER stress and the mechanisms through which ROS can be modulated.

The Peroxiredoxins (PRDXs) family, encompassing a crucial class of antioxidant enzymes, plays a vital role in cellular protection. This family includes several subtypes such as PRDX1-6, pivotal in maintaining the cellular redox state [28, 29]. PRDXs catalyze the conversion of cysteine to sulfonic acid, thus reducing peroxides. These enzymes are classified into three subfamilies based on the position of the cysteine residue in their catalytic reaction: typical 2-Cys (PRDX1-4), atypical 2-Cys (PRDX4), and 1-Cys (PRDX6) [30]. The PRDXs are instrumental in mitigating various diseases by scavenging ROS, thereby shielding cells from oxidative damage [31, 32]. Research has indicated that PRDXs modulate diverse phenotypes in cancer cells, including invasion, migration, epithelial-mesenchymal transition, and stem cell properties. They are also associated with tumor cells developing resistance to apoptosis [33]. Furthermore, PRDXs are involved in regulating the cellular redox balance, thus maintaining cellular homeostasis. They influence cell apoptosis by modulating ROS, Trx1, or ASK1/p38 signaling pathways, among other factors [34, 35]. In cancer cells, the expression levels of PRDXs members impact their resistance to anticancer drugs. For instance, increased levels of PRDX1, PRDX3, and PRDX6 in cancer types like breast cancer, ovarian cancer, and leukemia may lead to resistance to chemotherapy [36]. Therefore, the PRDXs family is essential for cell survival and functional maintenance. In this study, the PRDXs family is also critical in regulating cell apoptosis induced by BLM.

In this research, BLM, an anticancer drug, generates ROS, leading to the apoptosis of human cervical cancer SiHa cells. The study aims to elucidate the mechanism through which BLM induces cervical cancer cell death and the role of the PRDXs family as antioxidant enzymes in the process of BLM’s cytotoxic action on cancer cells.

## Result

### Bleomycin induces ER stress and apoptosis in cervical cancer cells

**Fig. 1:**
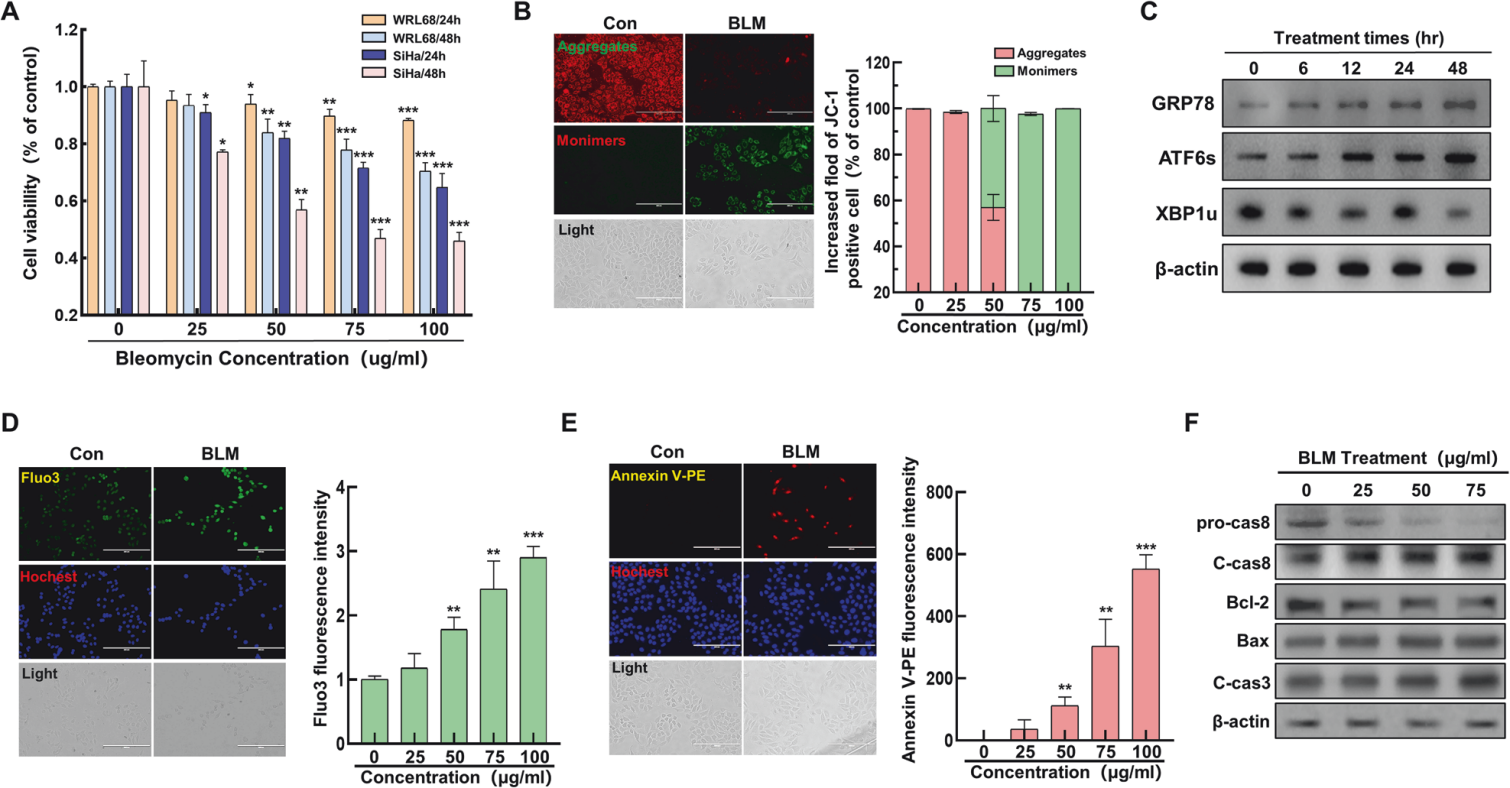
Impact of bleomycin on ER stress and cell death in cervical cancer cells. **A** Viability of WRL68 normal liver and SiHa cervical cancer cells following bleomycin exposure. **B** Analysis of mitochondrial membrane potential changes using JC-1 staining in response to bleomycin (concentrations: 0, 25, 50, 75, 100 µg/mL), with qualitative (left) and quantitative (right) assessments. **C** Western blot detection of ER stress markers in cells treated with bleomycin for varying durations (0, 6, 12, 24, 48 hours). **D** Evaluation of Ca2+ release in cells post-bleomycin treatment, examined through fluorescence microscopy (left) and quantitatively (right) across different dosages. **E** Assessment of cell apoptosis via Annexin-V staining after bleomycin treatment, visualized through fluorescence microscopy (left) and quantified (right) for each concentration level. **F** Western blot analysis of apoptosis-related proteins (pro-caspase8, cleaved-caspase8, Bcl2, Bax, cleaved-caspase3) post-treatment with various bleomycin concentrations (0, 25, 50, 75 µg/mL). Significance indicated by *P < 0.05; **P < 0.01; ***P < 0.001.

The study aimed to ascertain the impact of BLM on cell viability and its role in inducing ER stress and apoptosis in cervical cancer cells. WRL68 human normal liver cells and SiHa human cervical cancer cells were subjected to bleomycin treatment at varying concentrations (0, 25, 50, 75, and 100 µg/mL) for durations of 24 and 48 hours, followed by an MTT assay. The study results showed that bleomycin decreased the survival of SiHa cells in a manner that was dependent on both the dosage and duration of treatment (Fig. 1A). Further analysis involved assessing the effect of BLM on mitochondrial membrane potential in SiHa cells using JC-1 fluorescence staining. A noticeable dose-dependent decrease in mitochondrial membrane potential was observed with increasing BLM concentrations (Fig. 1B). To explore the link between BLM-induced mitochondrial damage and ER stress, SiHa cells were treated with BLM (75 µg/mL) over time, and the expression levels of ER stress-related proteins were evaluated using Western Blotting. The results indicated a gradual increase in ER stress-related proteins correlating with the duration of BLM treatment (Fig. 1C). Additionally, the study investigated the ER’s response to stress by measuring intracellular calcium ion levels through Fluo-3 fluorescence staining. A consistent increase in calcium ion levels was noted as the BLM concentration increased (Fig. 1D), suggesting that severe ER stress ensued following mitochondrial damage, leading ultimately to cell apoptosis. The extent of apoptosis was further examined using Annexin-V-PE fluorescence staining and immunoblotting techniques. The analysis demonstrated a significant escalation in the level of cell apoptosis proportional to the BLM treatment concentration, encompassing both mitochondrial-dependent and mitochondrial-independent pathways (Fig. 1E, F). In summary, the results conclusively indicate that BLM induces substantial ER stress in cervical cancer cells, leading to apoptosis through mitochondrial damage.

### Inhibition of PRDX I and II enhances bleomycin-induced ER stress

**Fig. 2:**
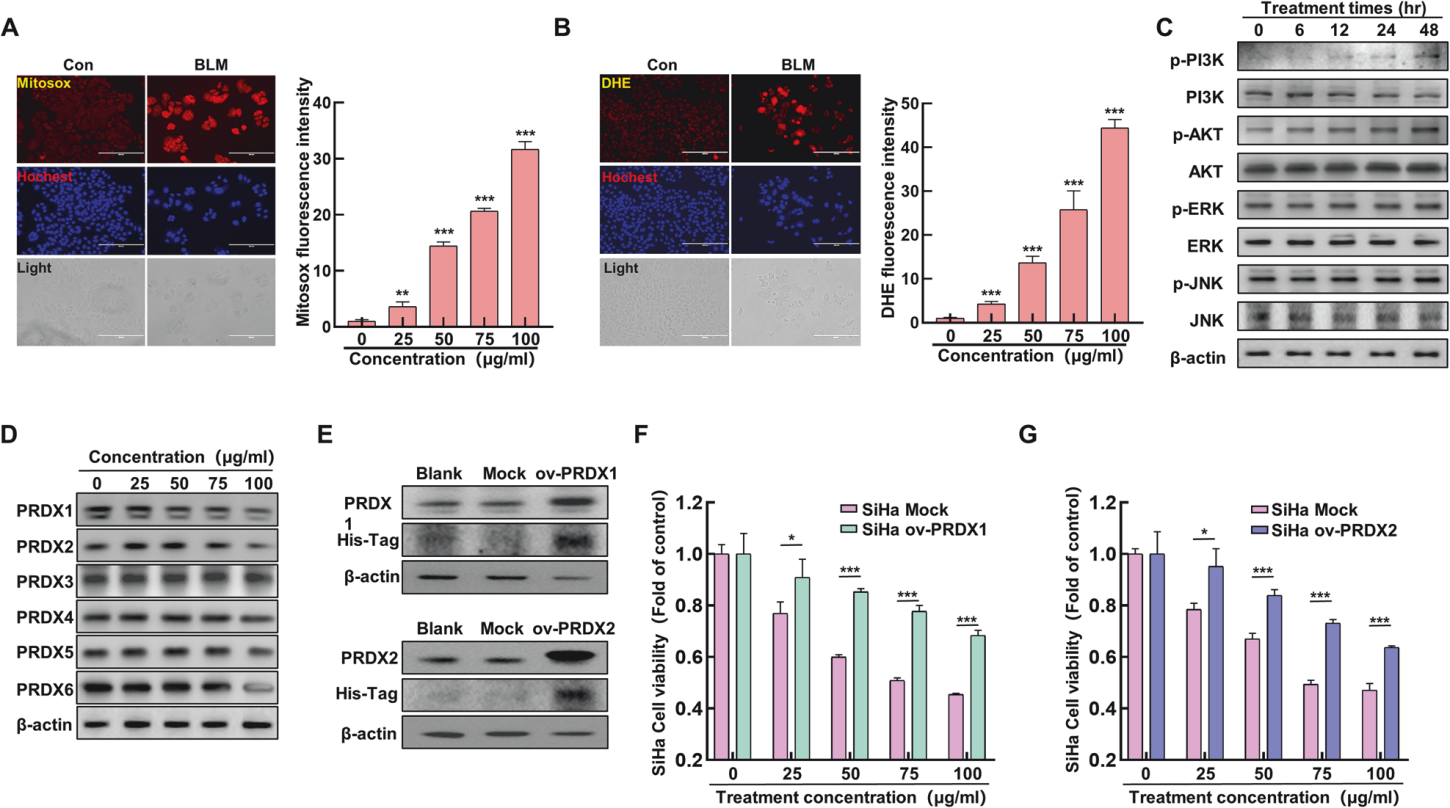
Impact of peroxiredoxin I and II inhibition on bleomycin-induced ER stress. **A** Mitosox staining to assess mitochondrial ROS in SiHa cervical cancer cells was treated with varying BLM concentrations (0, 25, 50, 75, 100 µg/mL); qualitative (left) and quantitative (right) analyses were performed using fluorescence microscopy. **B** DHE staining to quantify intracellular ROS levels at different BLM doses (0, 25, 50, 75, 100 µg/mL), analyzed qualitatively (left) and quantitatively (right) via fluorescence microscopy. **C** Western blot for PI3K/AKT and MAPK pathway proteins following BLM treatment over time (0, 6, 12, 24, 48 hours). **D** Western blot of PRDXs proteins post BLM treatment (0, 25, 50, 75, 100 µg/mL). **E** Construct SiHa cell lines overexpressing PRDX1 and PRDX2 and analyze the expression of PRDX1 and PRDX2 proteins using western blot. **F** Treat Mock and ov-PRDX1 SiHa cells with different concentrations of BLM (0, 25, 50, 75, 100 µg/mL), and analyze cell viability. **G** Treat Mock and ov-PRDX2 SiHa cells with different concentrations of BLM (0, 25, 50, 75, 100 µg/mL), and analyze cell viability. Significance indicated by **P < 0.01; ***P < 0.001.

In the previously discussed section, it was noted that BLM can lead to mitochondrial damage, commonly associated with increased levels of ROS in the mitochondria and within cells. To further investigate this, SiHa cells were treated with a concentration gradient of BLM, and the ROS levels within the mitochondria and cells were measured using Mitosox and DHE fluorescent staining. The results indicated a gradual increase in mitochondrial (Fig. 2A) and intracellular ROS levels (Fig. 2B) as the BLM concentration was elevated. Further analysis involved treating SiHa cells with BLM over time and assessing the expression levels of proteins in the MAPK and PI3K/AKT signaling pathways. Interestingly, while the MAPK pathway showed no significant changes, the PI3K/AKT pathway’s protein expression levels notably increased with the duration of BLM exposure (Fig. 2C). This suggests that ROS might activate the PI3K/AKT pathway, subsequently leading to ER stress. The role of the PRDXs family, known for its antioxidant properties, was then explored in the context of BLM-induced ER stress. Upon treating the cells with various concentrations of BLM, a significant decrease in the expression levels of PRDX1 and PRDX2 was observed (Fig. 2D), unlike the other PRDXs family proteins. We established SiHa cell lines overexpressing Mock, ov-PRDX1, and ov-PRDX2, and confirmed the successful overexpression of PRDX1 and PRDX2 using protein immunoblotting. The results indicated successful construction of SiHa cell lines overexpressing PRDX1 and PRDX2 (Fig. 2E). Subsequently, SiHa cell lines overexpressing Mock, ov-PRDX1, and ov-PRDX2 were treated with different concentrations of BLM, and their cell viability was assessed using MTT assay. The results showed that the cell viability of ov-PRDX1 and ov-PRDX2 groups decreased significantly with increasing BLM concentration. However, at the same concentration of BLM treatment, cell viability was notably higher compared to the Mock group (Fig. 2F, G). The results above indicate that BLM can elevate mitochondrial and intracellular ROS levels and activate the PI3K/AKT signaling pathway. Additionally, BLM can inhibit the expression levels of PRDX1 and PRDX2. Moreover, SiHa cells overexpressing PRDX1 show a significant resistance to BLM.

### Overexpression of PRDX I and II reduces bleomycin-induced ER stress in cervical cancer cells

**Fig. 3:**
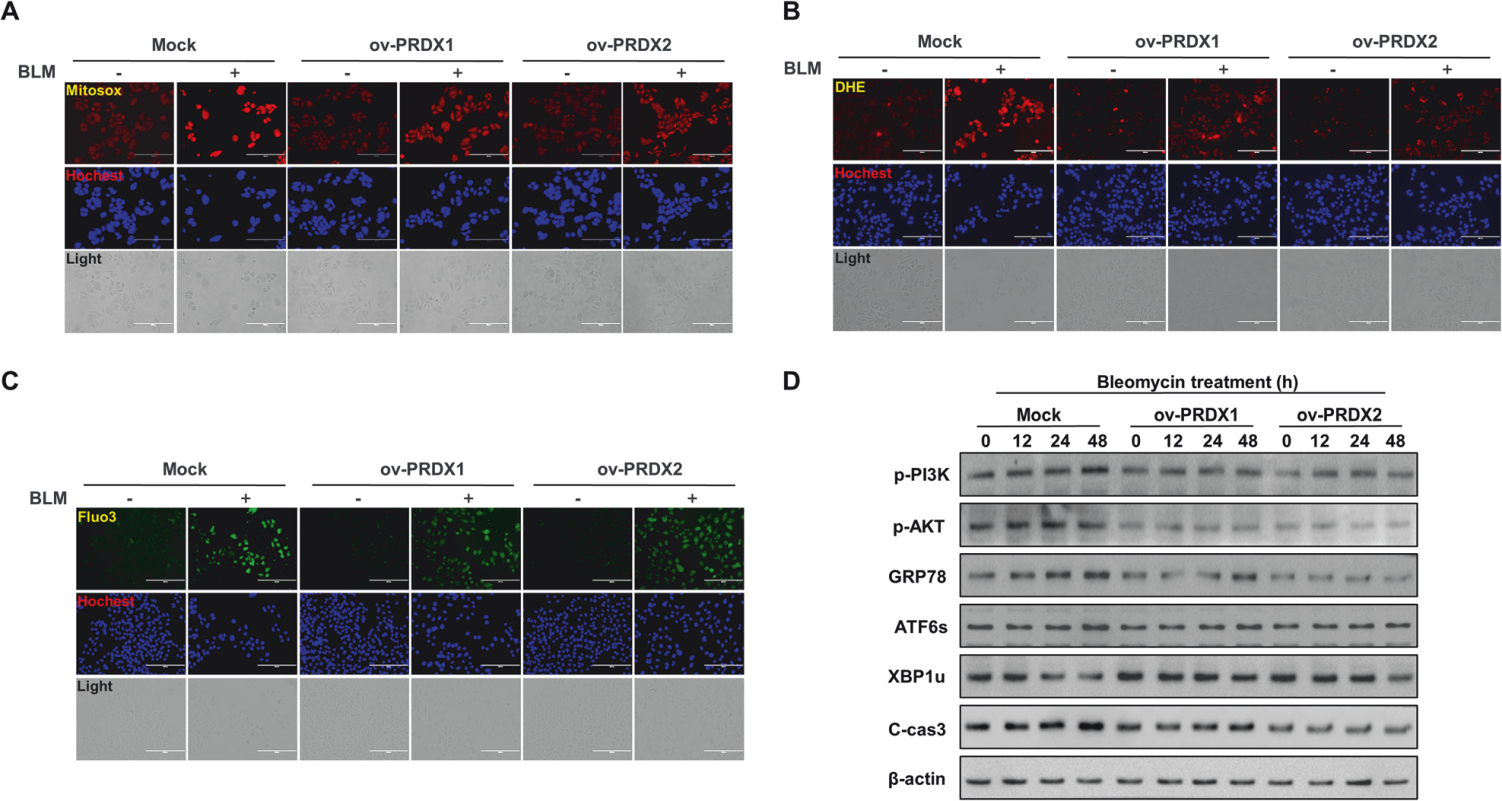
Reduction of bleomycin-induced ER stress by overexpression of peroxiredoxin I and II in cervical cancer cells. **A** Analysis of mitochondrial ROS levels using Mitosox staining in SiHa cells treated with 75 µg/mL BLM post Mock, ov-PRDX1, and ov-PRDX2, quantified by fluorescence microscopy. **B** Evaluation of intracellular ROS using DHE staining in SiHa cells treated with 75 µg/mL BLM following Mock, ov-PRDX1, and ov-PRDX2, measured through fluorescence microscopy. **C** Determination of intracellular calcium levels in SiHa cells treated with 75 µg/mL BLM after Mock, ov-PRDX1, and ov-PRDX2, using Fluo-3 staining and analyzed via fluorescence microscopy. **D** Western blot assessment of PI3K/AKT, ER stress, and apoptosis-related proteins in BLM-treated (0, 12, 24, 48 hours) SiHa cells, comparing Mock, ov-PRDX1, and ov-PRDX2 groups.

The study explored the significant roles of PRDX I and II (PRDX1 and PRDX2) in the context of BLM treatment in SiHa cervical cancer cells. Specifically, SiHa cells overexpressing PRDX1 and PRDX2 were treated with BLM at a concentration of 75 µg/mL, and the levels of ROS in the mitochondria and intracellular environment were assessed. The findings indicated that overexpressing PRDX1 and PRDX2 led to a substantial decrease in both mitochondrial and intracellular ROS levels when compared to the control (mock group), particularly under BLM treatment conditions (Fig. 3A, B). Moreover, the study measured the levels of intracellular calcium, a key indicator of ER stress. It was observed that the cells overexpressing PRDX1 and PRDX2 exhibited significantly lower levels of intracellular calcium compared to the mock group (Fig. 3C). Further investigations were conducted to understand the regulatory effects of PRDX1 and PRDX2 on ER stress. This involved treating the overexpressing cells with BLM over a time gradient and then analyzing the expression levels of proteins related to the PI3K/AKT signaling pathway, ER stress, and apoptosis. The results showed that in the groups overexpressing PRDX1 and PRDX2, the expression levels of PI3K/AKT signaling pathway-related proteins, ER stress-related proteins, and apoptosis-related proteins were markedly decreased compared to the Mock group (Fig. 3D). This outcome suggests that PRDX1 and PRDX2 play a pivotal role in mitigating ROS and reducing ER stress, particularly in the regulation of ROS induced by BLM treatment. This finding is critical in understanding the therapeutic potential of PRDX1 and PRDX2 in managing ER stress and apoptosis in cervical cancer cells treated with BLM.

### Knockdown of PRDX I and II enhances bleomycin-induced ER stress in cervical cancer cells

**Fig. 4:**
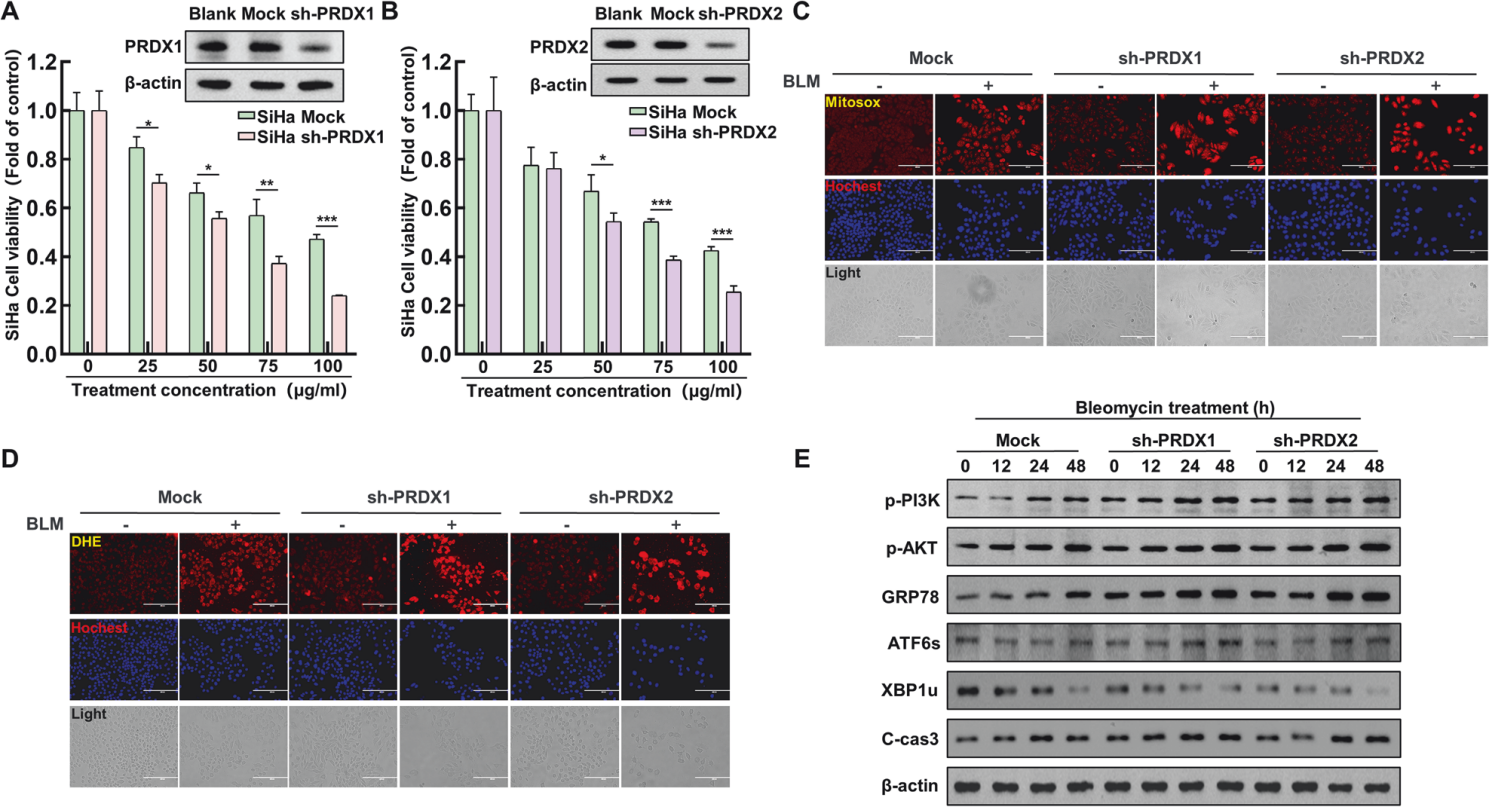
Enhanced ER stress in cervical cancer cells due to knockdown of peroxiredoxin I and II with bleomycin treatment. **A** Establishment of SiHa cell lines with PRDX1 knockdown, followed by Western blot analysis of PRDX1 expression. Cell viability was assessed in both Mock and sh-PRDX1 cells after treatment with varying BLM concentrations (0, 25, 50, 75, 100 µg/mL). **B** Generation of SiHa cell lines with PRDX2 knockdown and subsequent Western blot analysis of PRDX2 levels. Cell viability was evaluated in Mock and sh-PRDX2 cells post-treatment with different BLM concentrations (0, 25, 50, 75, 100 µg/mL). **C** Mitosox staining was conducted on Mock, sh-PRDX1, and sh-PRDX2 SiHa cells for quantitative mitochondrial ROS analysis using fluorescence microscopy, post BLM treatment (75 µg/mL). **D** DHE staining was performed on Mock, sh-PRDX1, and sh-PRDX2 SiHa cells for quantitative intracellular ROS assessment via fluorescence microscopy, following BLM treatment (75 µg/mL). **E** Western blot analysis of PI3K/AKT, ER stress, and apoptosis-related proteins in Mock, sh-PRDX1, and sh-PRDX2 SiHa cells after varying durations of BLM treatment (0, 12, 24, 48 hours). Significance indicated by *P < 0.05; **P < 0.01; ***P < 0.001.

This part of the study aimed to further elucidate the roles of PRDX1 and PRDX2 in cervical cancer treatment. SiHa cervical cancer cell lines were engineered with empty vectors, and specific knockdowns for PRDX1 and PRDX2 were created. The altered cells were subsequently subjected to different levels of BLM. Cell survival rates following BLM treatment were evaluated using an MTT assay. The results showed a significant decrease in survival rates following the knockdown of PRDX1 and PRDX2 (Fig. 4A, B). The study proceeded with treating SiHa cells with BLM (75 µg/mL) across three groups: mock, sh-PRDX1, and sh-PRDX2. The levels of ROS in mitochondria and intracellular environments were measured. It was observed that under BLM treatment, cells with PRDX1 and PRDX2 knockdown exhibited significantly higher levels of ROS both in the mitochondria and intracellularly compared to the mock group (Fig. 4C, D). Furthermore, the regulatory role of PRDX1 and PRDX2 on ER stress was investigated by treating the SiHa cells with a time gradient of BLM in the mock, sh-PRDX1, and sh-PRDX2 groups. The expression levels of proteins related to the PI3K/AKT signaling pathway, ER stress, and apoptosis were analyzed. The research revealed that silencing PRDX1 and PRDX2 resulted in a significant rise in the levels of proteins associated with the PI3K/AKT signaling pathway, ER stress, and apoptosis, compared to the Mock group. (Fig. 4E). These findings reinforce the critical roles of PRDX1 and PRDX2 in the modulation of ROS and their impact on ER stress in the context of BLM treatment. The knockdown of these proteins appears to exacerbate ER stress, highlighting their potential as therapeutic targets in managing ER stress and apoptosis in cervical cancer cells treated with bleomycin.

### PRDX I and II negatively influence survival in cervical cancer patients

**Fig. 5:**
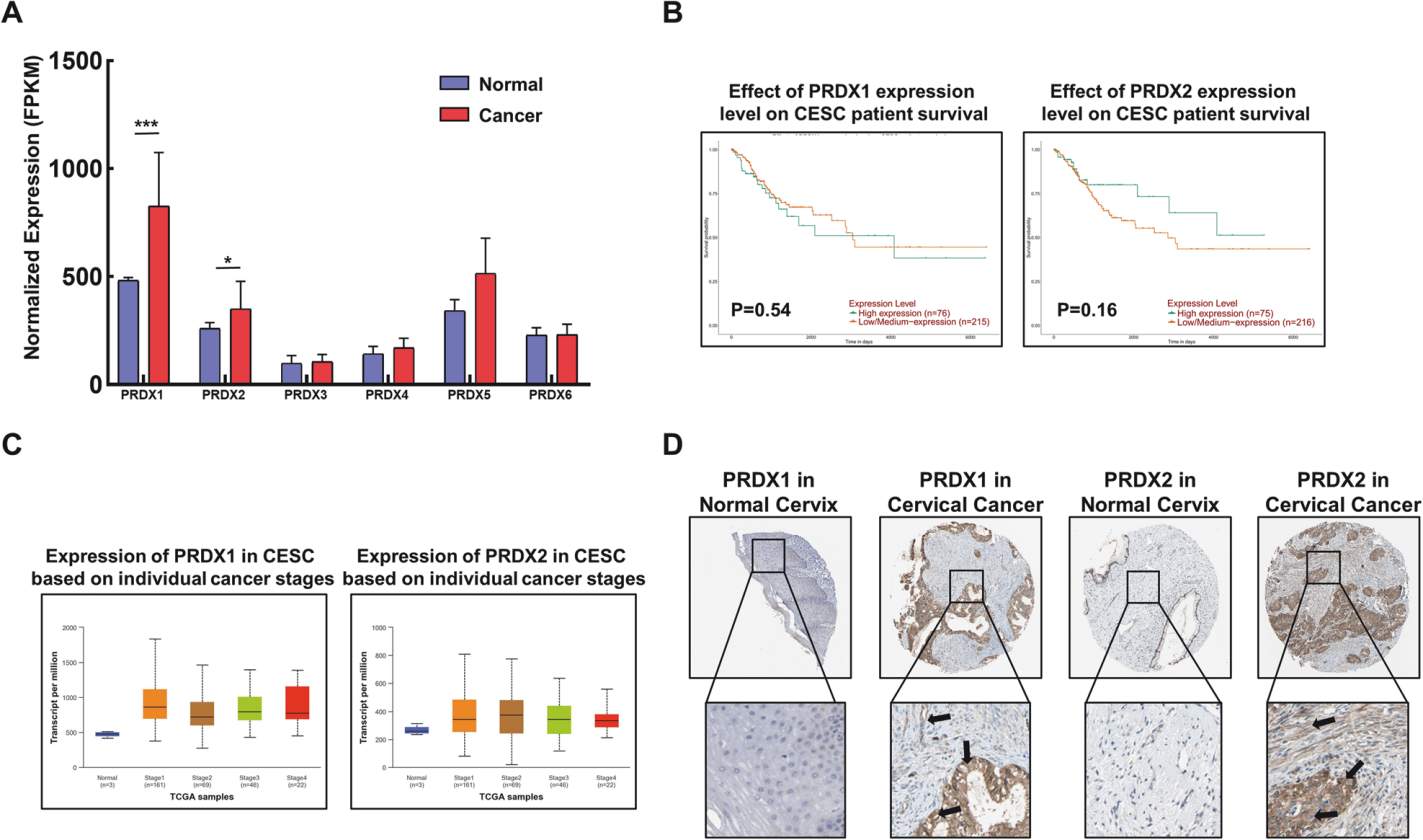
Role of peroxiredoxin I and II in cervical cancer patient survival. **A** Evaluation of PRDXs family expression in cervical cancer patients utilizing UALCAN database (https://ualcan.path.uab.edu/). **B** Prognostic survival analysis of PRDX1 and PRDX2 in cervical cancer using UALCAN. **C** Assessment of PRDX1 and PRDX2 expression across different stages of cervical cancer via UALCAN. **D** Comparative study of PRDX1 and PRDX2 expression in normal and cervical cancer tissues using Human Protein Atlas (https://www.proteinatlas.org/). Significance indicated by *P < 0.05; **P < 0.01; ***P < 0.001.

To investigate whether PRDX1 and PRDX2 play a crucial role in cervical cancer, an in-depth analysis was conducted to understand the role of PRDX1 and PRDX2 in cervical cancer. Utilizing UALCAN, a bioinformatics analysis platform, the expression levels of the PRDXs family in cervical cancer were examined. The analysis revealed that, compared to normal tissues, PRDX1 and PRDX2 expression levels were significantly higher in cervical cancer tissues (Fig. 5A). Further investigation into the impact of PRDX1 and PRDX2 on the prognosis and survival of cervical cancer patients was conducted, including their expression levels at various stages of the disease. The findings indicated no significant changes in expression levels across different stages, but a trend was observed where higher expression levels of PRDX1 and PRDX2 correlated with lower survival rates in patients (Fig. 5B). Moreover, the expression levels of PRDX1 and PRDX2 were consistently higher across different stages of cervical cancer (Fig. 5C). In addition, the expression of PRDX1 and PRDX2 in cervical tissues, including cancerous tissues, was studied using the Human Protein Atlas database. Consistent with the previous findings, levels of PRDX1 and PRDX2 were significantly higher in cervical cancer tissues in comparison to normal cervical tissues (Fig. 5D). The results above indicate that the expression levels of PRDX1 and PRDX2 are significantly elevated in both cervical cancer patients and cervical cancer tissues compared to normal levels.

### Inhibition of PRDX I and II enhanced the bleomycin anti-tumor property in xenograft mice model

**Fig. 6:**
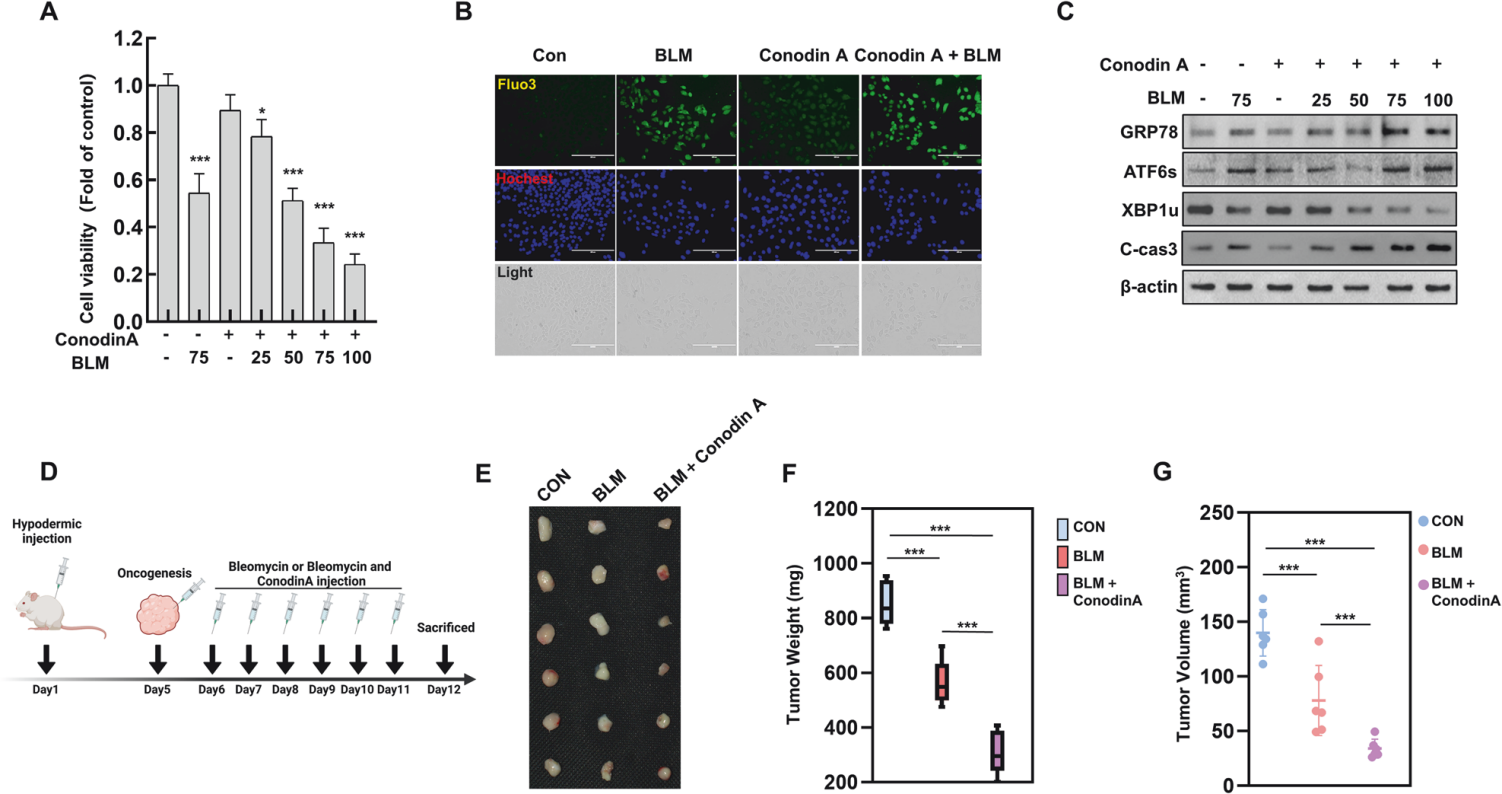
Enhanced anti-tumor effectiveness of bleomycin with conodin A in xenograft mice model. **A** Cell viability assessment with BLM (75 µg/mL) alone and BLM (0, 25, 50, 75, 100 µg/mL) combined with Conodin A. **B** Fluo-3 staining to measure intracellular Ca2+ levels following treatment with BLM (75 µg/mL) alone and combined with BLM (75 µg/mL) and Conodin A, with quantitative fluorescence microscopy analysis. **C** Western blot of ER stress proteins post-treatment with BLM (75 µg/mL) alone and in conjunction with BLM (0, 25, 50, 75, 100 µg/mL) plus Conodin A. **D** Detailed chronology of the experimental procedure. Initially, SiHa cervical cancer cells were subcutaneously injected into mice. On the fifth day, tumor formation was observed. Mice were treated with bleomycin (30 mg/mL/mouse) alone and in conjunction with Conodin A (5 mg/mL/mouse) over 7 days. Post-treatment, blood, liver, spleen, lung, kidney, and tumor tissues were collected for further analysis. **E** Post-tumor extraction, the tumors were photographed against a black backdrop to document size differences in the control, BLM, and BLM+Conodin A groups. **F** Following photography, tumors were weighed using an electronic balance to compare tumor weights across the control, BLM, and BLM+Conodin A groups. **G** Tumor dimensions were measured with a caliper, and volumes were calculated (length × width^2^) for each group: control, BLM, and BLM+Conodin A. Statistical significance denoted as *P < 0.05; ***P < 0.001.

**Fig. 7:**
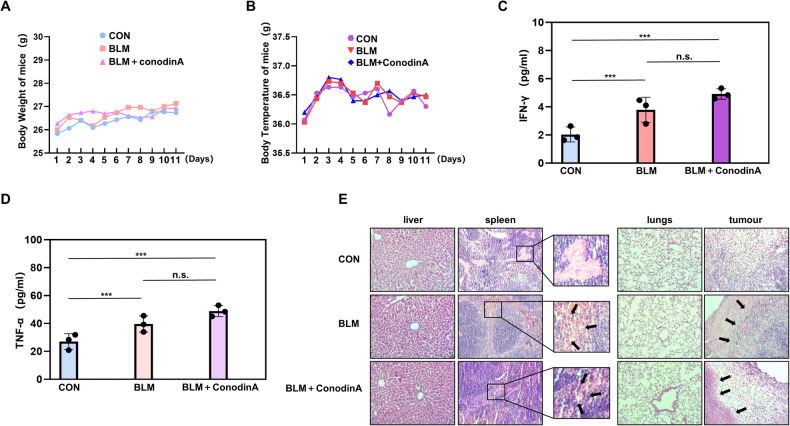
Analysis of serum cytokines and tissue effects in bleomycin and conodin A co-treated mice. **A** Monitoring and recording of body weight changes in control, BLM, and BLM+Conodin A groups from the day prior to cell injection till the day before euthanasia, using an electronic scale. **B** Temperature measurements of mice in control, BLM, and BLM+Conodin A groups, conducted with an electronic thermometer from the day before cell suspension injection to the day preceding euthanasia. **C** Serum from mice was isolated to evaluate IFN-γ levels using a specific detection kit. The concentration of IFN-γ was measured in three groups: control (Con), bleomycin only (BLM), and a combination of bleomycin with Conodin A (BLM + Conodin A). **D** Similarly, TNF-α levels in mouse serum were assessed using a dedicated detection kit. TNF-α concentrations were analyzed for the same three groups: Con, BLM, and BLM + Conodin A. **E** Tissue samples from the liver, spleen, lung, and tumor of mice in each group (Con, BLM, and BLM + Conodin A) underwent Hematoxylin and Eosin (H&E) staining. Microscopic examination of these stained tissues was performed to evaluate structural changes and damage, with results meticulously documented. Statistical significance across the groups is denoted as ***P < 0.001.

To assess the efficacy of BLM and the potential impact of PRDX1 and PRDX2 during treatment. When using BLM alone and in combination with Conodin A, the cell survival rate was measured using MTT. The results showed a significant decrease in cell survival rate when treated with BLM and Conodin A together compared to BLM treatment alone (Fig. 6A). Fluorescence imaging with Fluo-3 revealed a notable increase in intracellular calcium levels in the group treated with BLM and Conodin A compared to BLM treatment alone (Fig. 6B). Subsequently, changes in ER stress and cell apoptosis were examined using Western blot analysis. The results indicated that in the group treated with BLM and Conodin A, the protein expression levels of GRP78 and ATF6 were significantly elevated compared to BLM treatment alone, while the expression level of XBP1u was notably decreased, and the expression level of c-cas3 was markedly increased (Fig. 6C). Subsequently, we established subcutaneous tumor models by injecting cervical cancer SiHa cell suspension into the subcutaneous tissue of 18 mice. The mice were then categorized into three groups: a control group, a BLM monotherapy group, and a combination therapy group treated with both BLM and Conodin A (Fig. 6D). After a week of treatment, the tumors were excised for examination. It was observed that the tumors in the combination therapy group (BLM and Conodin A) were significantly smaller compared to those in other groups (Fig. 6E). Further analysis included measuring the weight and volume of the tumors, revealing that the combination therapy group showed a more pronounced reduction in both tumor weight and volume (Fig. 6F, G). This finding underscores the potent anti-tumor effect of BLM, which appeared to be enhanced by the addition of Conodin A. The injection of cell suspension was initiated, and the body weight and temperature of the mice were monitored (Fig. 7A, B). Serum samples were gathered from the mice to quantify the levels of INF-γ and TNF-α. The results indicated that these levels were considerably higher in the BLM-treated group compared to the control group, and even higher in the group receiving the combination therapy (Fig. 7C, D). Histopathological analysis through hematoxylin and eosin (H&E) staining of the liver, spleen, lungs, and tumor tissues from the mice showed no significant differences in the liver, spleen, and lungs across the three groups, suggesting a lack of toxic side effects from the treatment. In contrast, tumor tissues from the BLM monotherapy group displayed smaller and more regular cell nuclei, along with a denser structure, as opposed to the control group. The combination therapy group exhibited even more regular cell nuclei and denser tumor tissue structure (Fig. 7E). This pattern suggests that BLM treatment effectively inhibited tumor growth, and this effect was further enhanced by the addition of Conodin A.

## Discussion

Cervical cancer, a prevalent gynecological malignancy, necessitates effective therapies with minimal adverse effects to enhance patient outcomes. This study pioneers in assessing the role of the PRDXs family in cervical cancer treatment, a previously unexplored area. The research demonstrates that PRDX1 and PRDX2 modulate apoptosis in cervical cancer cells through the PI3K/AKT pathway-mediated ER stress, facilitated by the clearance of ROS. These findings underscore the critical role of PRDX1 and PRDX2 in apoptosis regulation within cervical cancer cells.

Bleomycin, a chemotherapy agent, exhibits diverse cytotoxic mechanisms in cancer treatment. It has been employed effectively in various cancers, including through injection, followed by electrochemical therapy in melanoma and head and neck cancer, as demonstrated in existing studies [37]. Additionally, its use in ovarian germ cell tumors shows that reduced dosages do not adversely affect patient survival [9]. A key mechanism of bleomycin’s action involves the generation of ROS, which occurs via pathways like mitochondrial damage and oxidative stress imbalance [38, 39]. Our investigation into the effects of bleomycin on human cervical cancer SiHa cells revealed a significant increase in both mitochondrial and intracellular ROS levels. This observation suggests that bleomycin’s anticancer efficacy may be partly attributed to its ability to elevate mitochondrial ROS, thereby initiating a sequence of events leading to cellular demise.

ROS, crucial oxidants found both inside and outside cells, and ion channels play a crucial role in maintaining cellular balance. They oversee cell communication, programmed cell death, and cell growth [40, 41]. Cancer cells often exhibit high ROS levels but also possess some resistance. However, excessive ROS can still damage these cells [42]. In this study, BLM treatment of cervical cancer SiHa cells led to increased intracellular ROS, suggesting that BLM’s cytotoxicity might partly stem from ROS generation. The PRDXs family, key antioxidant enzymes, primarily scavenges ROS [43]. Beyond this, PRDXs also regulate intracellular signaling pathways and are implicated in diseases like tumors and neurodegenerative disorders [44–47]. In our experiment, PRDX1 and PRDX2 emerged as critical in managing BLM-induced ER stress. Cells treated with BLM and Conodin A showed heightened ER stress. Overexpression of PRDX1 and PRDX2 mitigated apoptosis caused by BLM, while their knockdown had the opposite effect. This suggests that modulating PRDX1 and PRDX2 expression could enhance BLM’s efficacy against cervical cancer. Notably, BLM treatment reduced PRDX1 and PRDX2 levels in normal SiHa cells, indicating its potential impact on antioxidant enzymes, though the exact mechanism needs further exploration.

It is worth noting that in the xenograft mice experiments, we detected the serum IFN-γ and TNF-α after being treated with BLM alone and BLM combined with Conodin A. IFN-γ and TNF-α are crucial immunoregulatory factors that can be utilized in cancer treatment or directly inhibit tumor cell proliferation [48, 49]. In this study, there were no significant differences in the serum IFN-γ and TNF-α between the group treated with BLM alone and BLM combination with Conodin A. This may indicate that there are no side effects of conodin A on in vivo treatment and exhibits the synergy effects with BLM treatment of cervical cancer. Moreover, according to our results, we can find that Prdx1 and Prdx2 exhibit similar functions on BLM-induced cell apoptosis. It maybe because of that the Prdx1 and Prdx2 both belong to the classical 2-Cys PRDX family, and they are localized mainly in the cytoplasm, almost the same molecular weight, and scavenges similar level of the ROS in the cells [31, 44]. However, we have no direct evidences to improve it in the present study. This point should be further studied in our further researches.

ROS can directly oxidize proteins, affecting their structure [50]. Proteins, crucial biomolecules, perform diverse functions like structural support and enzyme activity [51–54]. The ER, vital for protein folding and repair, can be disrupted by increased ROS, resulting in protein misfolding and ER stress. In treating cervical cancer, ROS production leads to misfolded proteins, triggering the ER to degrade these proteins. The ER employs pathways like PERK, ATF6, and IRE1 during stress [55–59]. Persistent severe ER stress activates pro-apoptotic factors, leading to cell death [60]. The ER stress-related pathway, marked by increased GRP78 expression, indicates a strong link between ROS and ER stress [61]. BLM induces ER stress primarily through the generation of ROS. BLM can combine with molecular oxygen and ferrous ions to produce free radical substances such as O^2−^, hydroxyl radicals, and ferric ions. The ROS produced by bleomycin could also induced the DNA damage, protein misfolding, ion channel changes, and these phenomenon could also increase the ER stress [62]. The PERK pathway, known for inhibiting protein synthesis, was not initially detected, it likely trends with other ER stress proteins, warranting further study. This study suggests that managing ER stress by augmenting ROS could be an effective strategy for cervical cancer treatment.

In this research on treating human cervical cancer SiHa cells with BLM, a notable activation of the PI3K/AKT signaling pathway was observed, alongside the MAPK pathway. The PI3K/AKT pathway is vital in cancer biology, influencing cell proliferation, survival, and metabolism [63]. In this research on treating human cervical cancer SiHa cells with BLM, a notable activation of the PI3K/AKT signaling pathway was observed, alongside the MAPK pathway. The PI3K/AKT pathway is vital in cancer biology, influencing cell proliferation, survival, and metabolism [64]. Intriguingly, in our study, PI3K/AKT pathway activation by BLM treatment led to apoptosis in cancer cells. This finding suggests a complex role of the pathway, diverging from its usual function in promoting tumor cell survival. Moreover, the UPR under ER stress is a mechanism initially aimed at preserving cellular homeostasis. However, excessive stress can overwhelm the system, leading to apoptosis [65]. In our study, the link between PI3K/AKT pathway activation and ER stress was indicated through the modulation of PRDX1 and II, though direct evidence of this relationship is yet to be established. This indicates a potential area for further exploration to understand the intricate connections between these pathways in the context of cervical cancer treatment. The research results emphasize the pivotal roles of PRDX1 and PRDX2 in the treatment of cervical cancer using BLM. The administration of BLM results in the generation of ROS, which in turn induces ER stress and leads to apoptosis in cancer cells. However, PRDX1 and PRDX2 serve as ROS scavengers during this process, mitigating ER stress and consequently enhancing the survival rate of SiHa cells. This suggests that targeting PRDX1 and PRDX2, possibly through the use of inhibitors, might amplify the anti-cancer efficacy of BLM in cervical cancer therapy. This strategy could potentially provide a more effective approach to combat cervical cancer, emphasizing the significant therapeutic potential of PRDX1 and PRDX2 modulation in conjunction with BLM treatment.

**Fig. 8:**
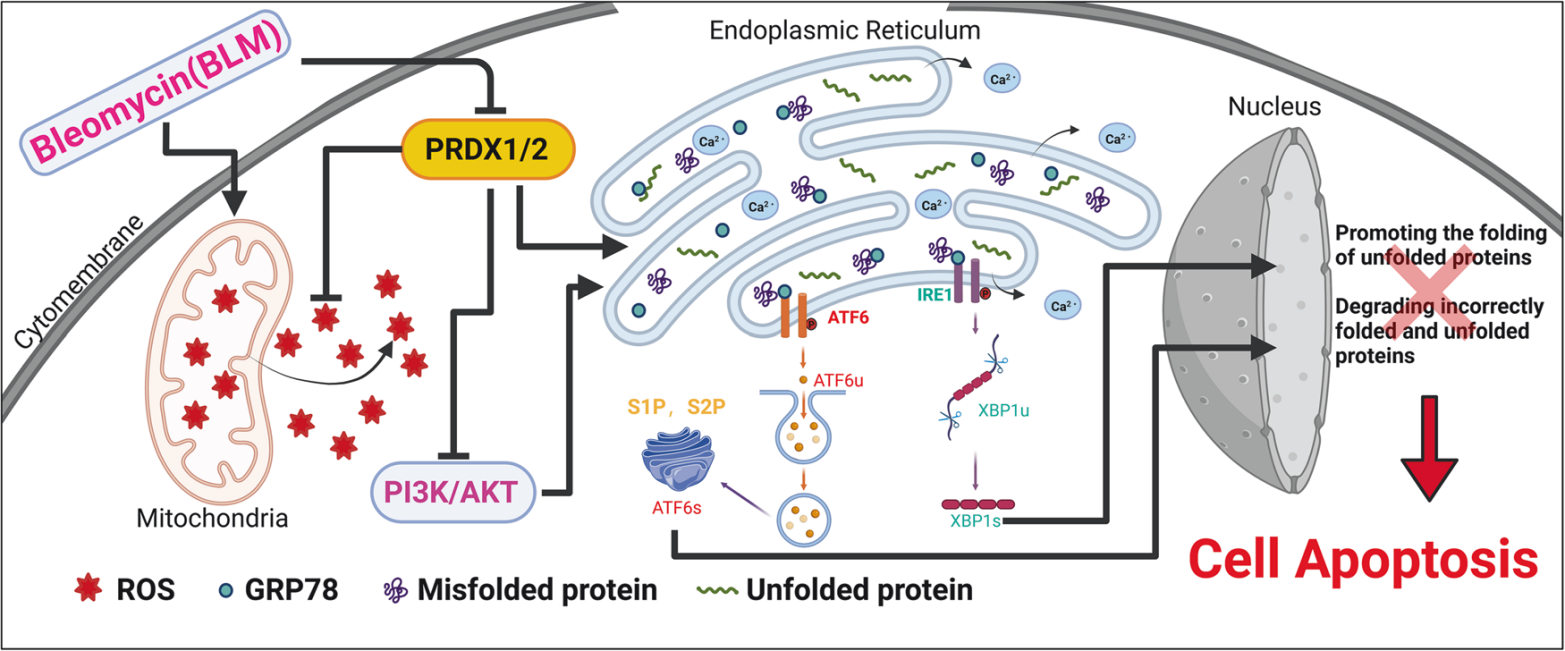
Mechanism diagram. This diagram illustrates the mechanistic pathway by which bleomycin induces apoptosis in cervical cancer cells. It demonstrates the involvement of Peroxiredoxin I and II in modulating the levels of reactive oxygen species (ROS) and their impact on endoplasmic reticulum (ER) stress, subsequently leading to cell apoptosis. The diagram also highlights the role of the PI3K/AKT signaling pathway in this process.

In conclusion, this study concludes that BLM treatment in cervical cancer cells increases both mitochondrial and intracellular ROS levels, causing mitochondrial damage. This leads to ER stress, culminating in cell apoptosis. PRDX1 and 2 play a significant role in this process by mitigating ER stress. They accomplish this by scavenging ROS and inhibiting the PI3K/AKT signaling pathway. Conversely, when PRDX1 and 2 are suppressed, ER stress intensifies, further inducing cell apoptosis. This intricate interplay between BLM, ROS, PRDX1 and 2, and the ER stress response reveals a complex mechanism underlying cervical cancer cell apoptosis, offering potential targets for therapeutic intervention (Fig. 8).

## Methods

### Cell culture

Cervical cancer SiHa cells, procured from Saibaikang Biotechnology Co., Ltd., Shanghai, China, were initially thawed at −150 °C. Following rapid thawing, the cells were transferred to a 15 mL centrifuge tube and mixed with 9 mL of Dulbecco’s Modified Eagle’s Medium (DMEM, Cytiva, Marlborough, MA, USA), supplemented with 10% fetal bovine serum (FBS, Solarbio Science and Technology Co., Ltd., Beijing, China) and 1% penicillin/streptomycin (P/S, Solarbio Science and Technology Co., Ltd., Beijing, China). The mixture was then centrifuged at 5000 rpm for 3 min Post-centrifugation, the supernatant was discarded, and the cells were resuspended in 6 mL of the culture medium. This cell suspension was subsequently transferred to a 10 cm culture dish. The container was gently agitated in all directions to ensure an even distribution of cells and then placed in a cell culture incubator maintained at 37 °C with 5% CO_2_. The culture medium was refreshed every 24 hours. Upon reaching over 90% confluency, the cells were washed with Phosphate-buffered saline (PBS, Cytiva, Marlborough, MA, USA) and treated with 1 mL of trypsin/EDTA solution (TE, Solarbio Science and Technology Co., Ltd., Beijing, China) for 3 minutes to facilitate digestion. This process was halted by adding 9 mL of culture medium. The cells were then thoroughly detached from the bottom of the dish, transferred to a 15 mL centrifuge tube, and centrifuged. After discarding the supernatant, the cells were resuspended in 6 mL of culture medium. Two 10 cm culture dishes were prepared, each receiving 3 mL of the cell suspension, supplemented with culture medium to a final volume of 10 mL. The dishes were thoroughly mixed and then placed back in the incubator. Post-centrifugation, the supernatant was removed, and the cells were resuspended in 1 mL of culture medium, followed by the addition of 1 mL of freezing solution (a 1:1 mixture of FBS and dimethyl sulfoxide (DMSO, Sigma-Aldrich, Merck KGaA, Darmstadt, Germany)). This mixture was then aliquoted into two cryovials, which were duly labeled and stored in a cryobox at −80 °C. After 24 hours, the cryovials were transferred to a −150 °C freezer for long-term storage.

### MTT assay

Cultivate well-grown cells, then centrifuge them post-digestion, allocating 8 × 10^3^ cells to each well of a 96-well plate for further cultivation. Once the cells fully adhere to the well surfaces, replace the original culture medium with a starvation medium containing 1% FBS and incubate for 1 hour. Proceed with drug treatment using varying concentrations of BLM (0, 25, 50, 75, and 100 µg/mL for 24 and 48 hours) sourced from Thermo Fisher Scientific, Inc., Waltham, MA, USA. Post-treatment, introduce 10 µL of 3-(4,5-dimethylthiazol-2-yl)-2,5-diphenyltetrazolium bromide (MTT, Amresco, VWR Life Science, Radnor, PA, USA) to each well and incubate in the culture chamber for 2 hours. Following this, discard the supernatant, add 100 µL of DMSO to each well to dissolve the formed formazan crystals, and allow it to react for 10 minutes. Finally, measure the optical density (OD) at 590 nm using an enzyme-linked immunosorbent assay (ELISA) reader.

### Fluorescence staining

After optimal cultivation, digest and centrifuge the cells at 5000 rpm for 3 minutes. Discard the supernatant, resuspend in 6 mL of culture medium, and transfer to a 24-well plate, adding 8 × 10^4^ cells to each well. Post-adherence, replace the existing culture medium with a medium containing 1% FBS for starvation, lasting 1 hour. Administer BLM treatment in varying concentrations. Subsequently, perform Fluo-3 staining for intracellular calcium ions (Invitrogen, Eugene, Oregon, USA), Annexin-V-PE staining for apoptosis assessment (Solarbio Science and Technology Co., Ltd., Beijing, China), Mitsox staining for mitochondrial intracellular ROS, JC-1 staining for mitochondrial membrane potential analysis, and DHE staining for intracellular ROS. Concurrently, stain the nucleus with Hoechst (Mitsox, JC-1, DHE, and Hoechst, Solarbio Science and Technology Co., Ltd., Beijing, China). Following the staining process, add 500 µL of PBS to each well for observation and recording under a fluorescence microscope.

### Western blot

The cells were treated with colistin at various concentrations and durations. Following treatment, the cell culture dishes were washed with PBS, and the wash solution was discarded. Cell lysis was performed to extract proteins, incorporating phosphatase and protease inhibitors into the lysis buffer. The protein concentration was quantified and subsequently mixed with 5× Buffer. The proteins were separated using SDS-PAGE gel electrophoresis and then transferred onto a nitrocellulose membrane (Millipore, Massachusetts, USA). For blocking, 5% skim milk was used. The primary antibodies, sourced from Santa Cruz Biotechnology, Inc., Dallas, TX, USA, were incubated with the membrane at room temperature for one hour or overnight at 4 °C. This was followed by incubation with horseradish peroxidase-conjugated goat anti-mouse IgG or anti-rabbit IgG (Sigma-Aldrich, Merck KGaA, Darmstadt, Germany). The final step involved visualization using a chemiluminescence imaging system.

### Bioinformatics analysis

The expression levels of the PRDXs family in cervical squamous cell carcinoma, including their expression at different stages and their impact on prognosis and survival rates, were analyzed using the UALCAN online database (https://ualcan.path.uab.edu/). The results from this analysis were downloaded for further use. In addition, the distribution of the PRDXs family in cervical cancer tissues was investigated using the Human Protein Atlas database (https://www.proteinatlas.org/). This comprehensive analysis aimed to elucidate the role and significance of the PRDXs family in the context of cervical cancer.

### Lentiviral transfection

SiHa cells were seeded into six culture dishes, each with a diameter of 6 cm. The experimental setup included various groups: the blank group, the empty overexpression group, the PRDX1 overexpression group, the PRDX2 overexpression group, and the PRDX1 and PRDX2 knockdown groups, using reagents supplied by GenePharma, Jiangsu, China. The cells were cultured until they reached approximately 30% confluence. Upon complete adherence of the cells, an appropriate volume of lentiviral solution, in accordance with the concentration of the purchased lentiviral vector, was added to the culture dishes. Additionally, a lentiviral enhancer was included to facilitate the construction of the following cell lines: blank knockdown SiHa, PRDX1 overexpression SiHa, PRDX2 overexpression, PRDX1 knockdown, and PRDX2 knockdown.

The cells were cultured continuously for three days. Post-cultivation, puromycin was used for cell selection in the three designated culture dishes. Once the cells in the blank group were entirely eliminated, the surviving cells from the blank vector control group, the PRDX1 overexpression group, and the PRDX2 overexpression group were transferred to 3.5 cm culture dishes. This step was undertaken to expand the cell populations for further experimental procedures.

### in vivo experiment

The animal study was conducted following approval from the Animal Ethics Committee of Heilongjiang Bayi Agricultural University. Mice were maintained in clean animal facilities at Heilongjiang Bayi Agricultural University, adhering to a 12:12 light/dark cycle and provided with standard nutrition. Adult mice were bred through natural mating processes.

For the experimental procedure, SiHa cervical cancer cells were cultured, with 1 × 10^7^ cells being resuspended in 100 µL of PBS buffer. Balb/c mice, aged between 6 and 8 weeks, were selected for the study. The backs of these mice were depilated to expose the skin for injections. Each mouse was subcutaneously injected with 1 × 10^7^ SiHa cells using a 1 mL syringe. Following the formation of subcutaneous tumors, the mice were divided into three groups for drug administration: a control group, a treatment group receiving BLM at a dose of 30 mg per mouse, and a combination treatment group administered with both BLM (30 mg per mouse) and Conodin A (5 mg per mouse). The treatment duration lasted for 7 days. Post-treatment, the mice were euthanized under anesthesia. Subsequently, samples, including blood, liver, spleen, lungs, kidneys, and subcutaneous tumor tissues, were collected for analysis. The volume and weight of the subcutaneous tumor tissues were measured before preservation in formalin, alongside other collected tissues, for further experimental investigations. The conduct of these animal experiments was in strict compliance with the ethical guidelines set by Heilongjiang Bayi Agricultural University.

### Detection of serum TNF-α and IFN-γ in mice

Post blood collection from the mice, the samples were allowed to stand for 2 hours before being centrifuged at 5000 rpm for 20 minutes. This process facilitated the separation and retrieval of the upper serum layer. The levels of IFN-γ and TNF-α in the serum were determined using ELISA kits (IFN-γ kit from R&D Systems, Inc., Minneapolis, MN, USA and TNF-α kit from Invitrogen, Eugene, Oregon, USA). The procedure involved adding specific reagents as per the kit instructions, followed by a washing step, and then the addition of a stop solution. Finally, an enzyme immunoassay analyzer was employed to measure absorbance values. The concentrations of IFN-γ and TNF-α were calculated based on the established standard curve.

### H&E stains

Upon collection, mouse tissues were fixed in formalin. Before proceeding with H&E staining, the fixed tissues were dehydrated, embedded in paraffin at −20 °C, and sectioned into 5 µm slices using a tissue slicer. These tissue slices were then placed in warm water at 42 °C to flatten them before being transferred onto glass slides. Each slide was labeled, and the tissues were stained using H&E. Post-staining, the slides were sealed using a xylene-diluted resin and left to air dry for one day. The stained tissues were then observed and recorded under an optical microscope.

### Statistical analysis

The collected data were analyzed employing a repeated measures analysis of variance, complemented by independent sample *t* tests to compare the means between different groups. All statistical analyses were carried out using SPSS software, version 19.0. The significance of differences was determined based on the *p* value, with a threshold set for statistical significance. Specifically, differences were considered statistically significant (*P < 0.05; **P < 0.01; and ***P < 0.001).

### Supplementary information

WB original data

## Data Availability

The data that support the findings of this study are available from the corresponding author upon reasonable request.
