# Supplementary material for: Peroxiredoxin I and II as novel therapeutic molecular targets in cervical cancer treatment through regulation of endoplasmic reticulum stress induced by bleomycin
Source: Cell Death Discov. 2024 May 31;10:267. doi: 10.1038/s41420-024-02039-7 (PMC11143287; doi:10.1038/s41420-024-02039-7)
Supplement: Supplementary file 1 — WB original data [file 41420_2024_2039_MOESM1_ESM.pdf]

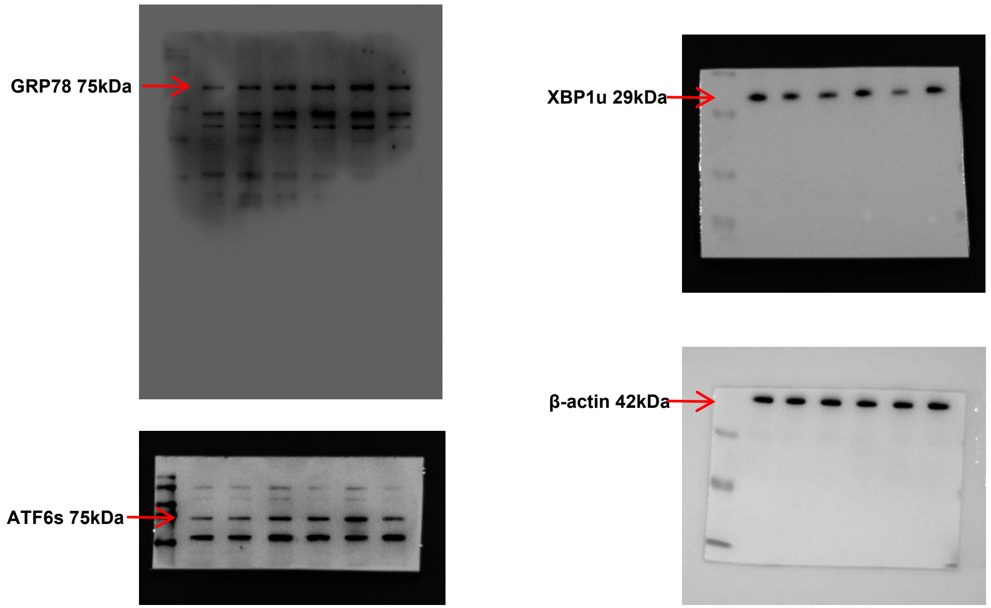

Fig1. C BLM(0,6,12,24,48(h))

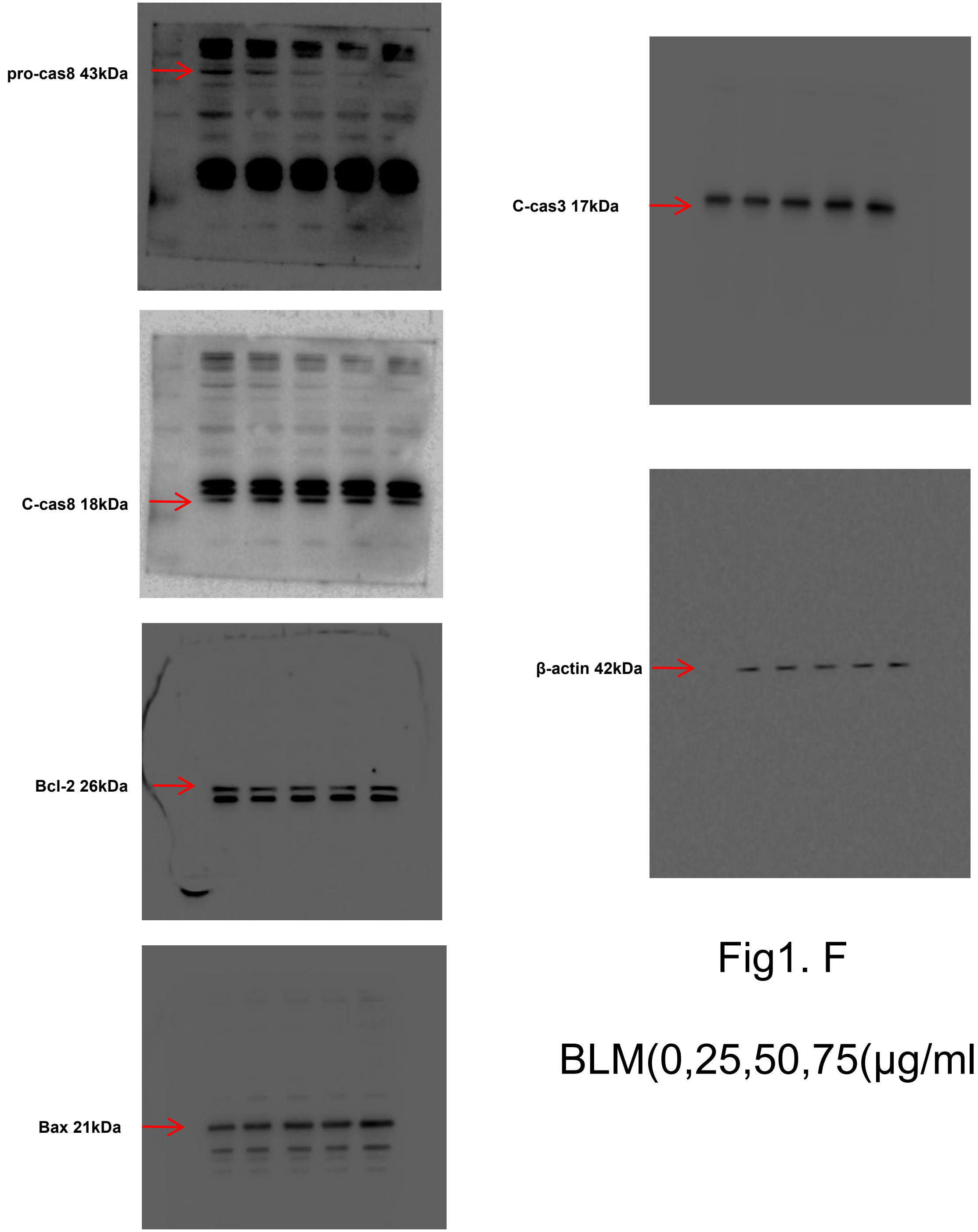

Fig1. F

BLM(0,25,50,75(μg/ml))

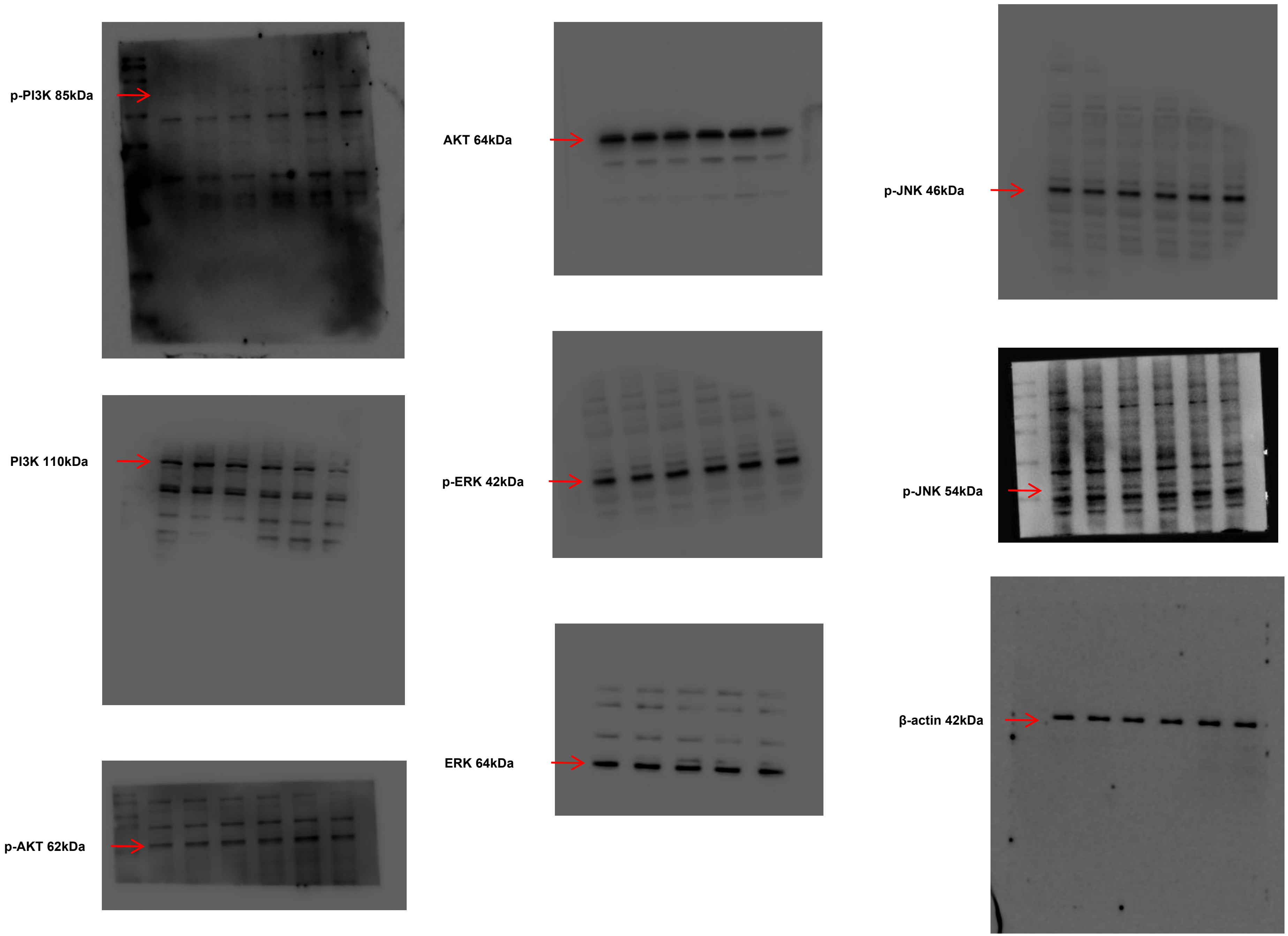

Fig2. C

BLM(0,6,12,24,48(h))

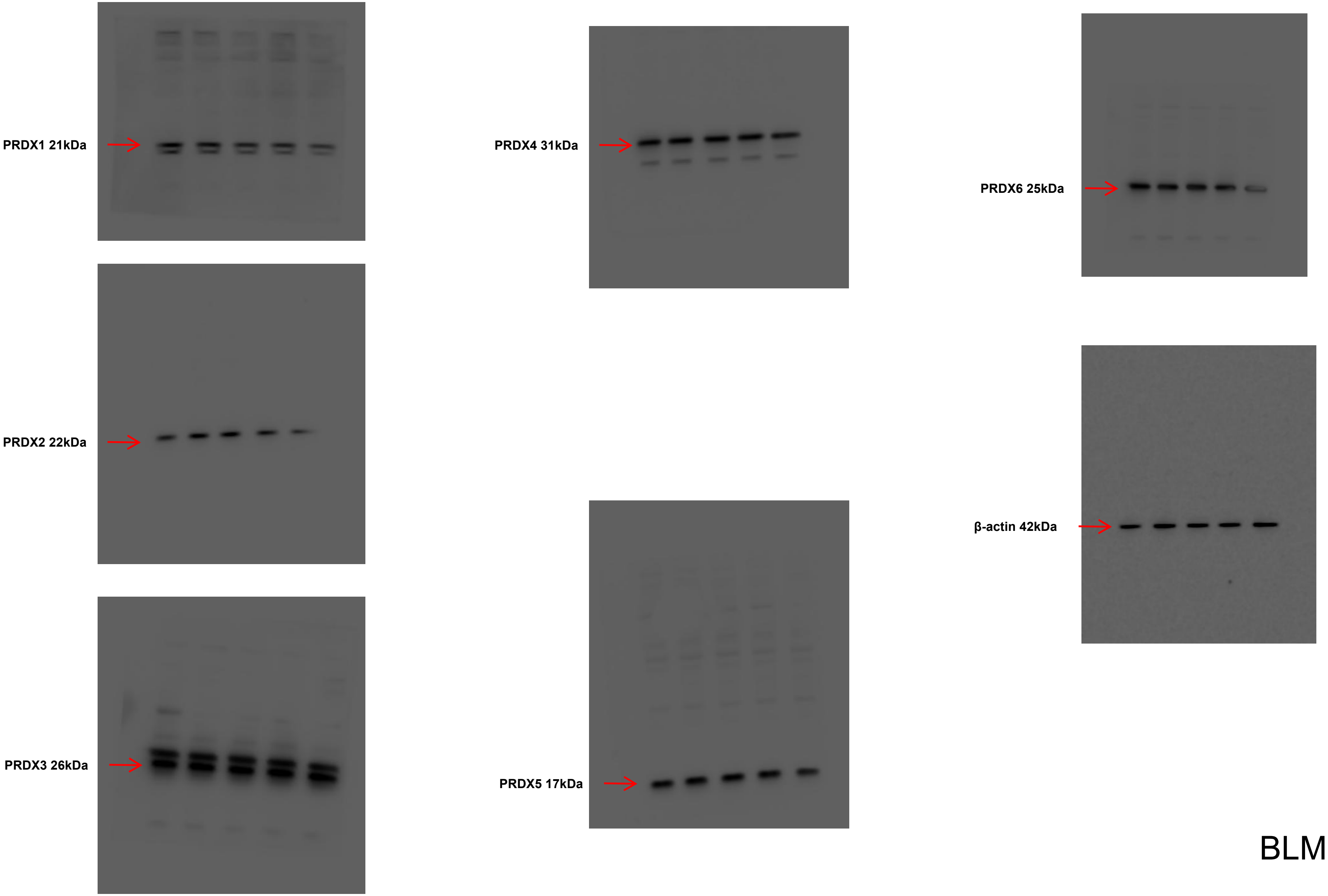

Fig2. D

BLM(0,25,50,75(μg/ml))

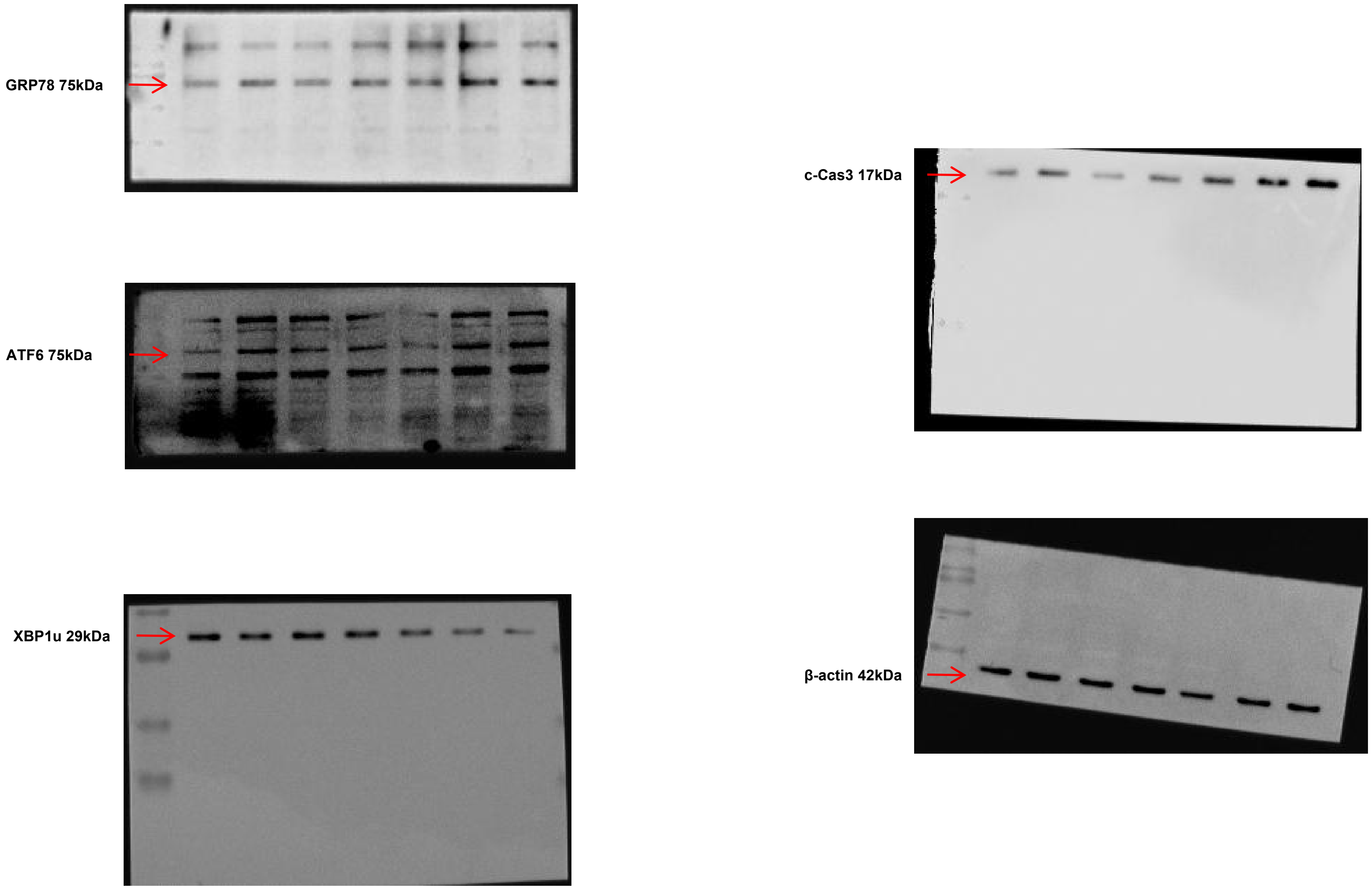

Fig2. F

BLM(0,75(μg/ml))(Conodin A (5μm),Conodin A + BLM 25,Conodin A + BLM 50,Conodin A + BLM 75(μg/ml))

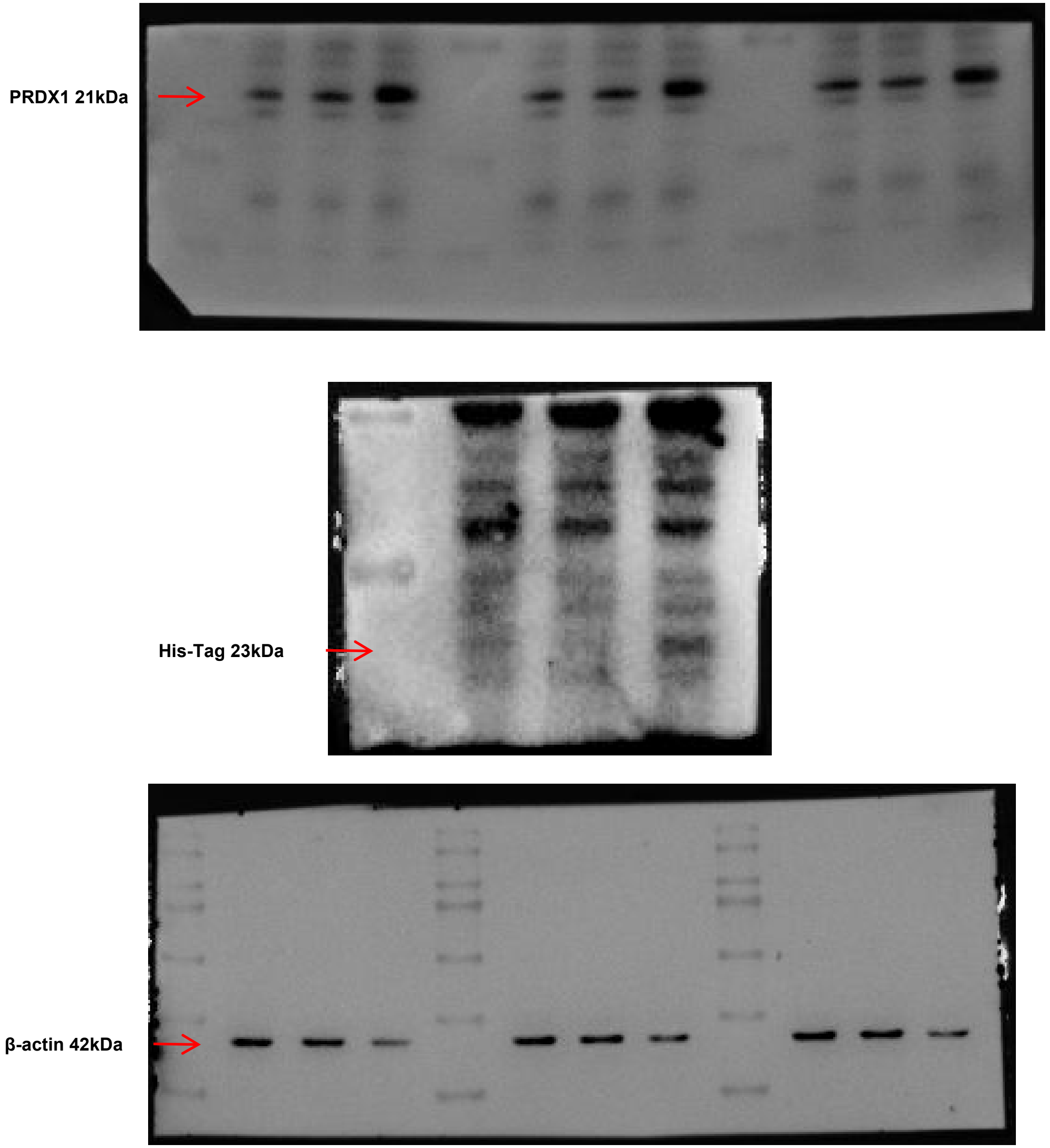

Fig3. E

SiHa (Blank,Mock,ov-PRDX1)

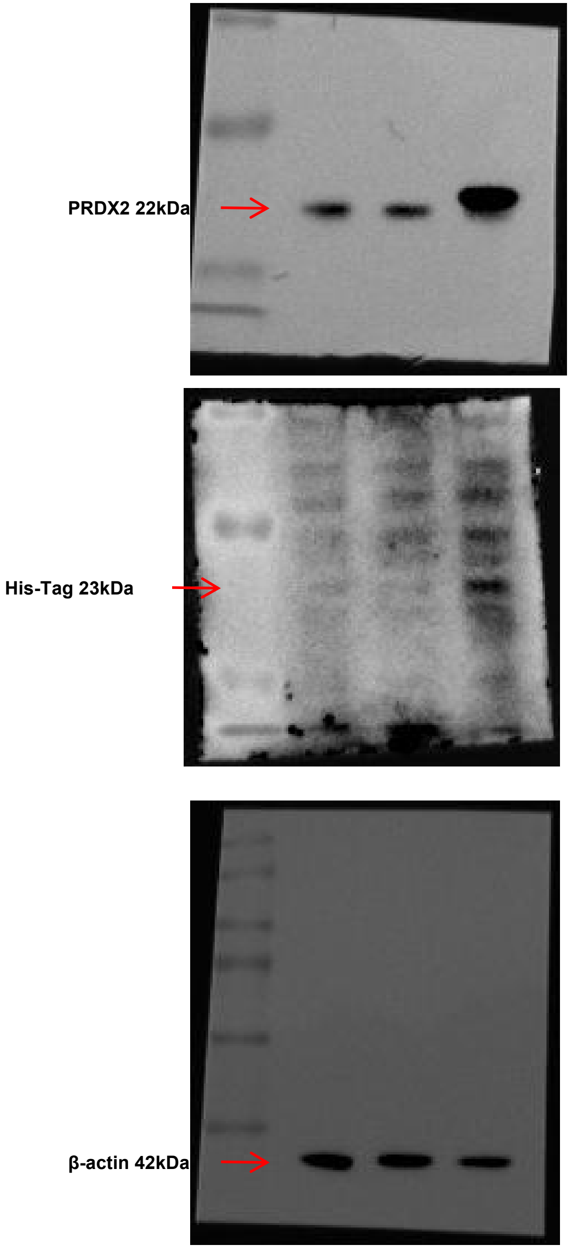

Fig3. F

SiHa (Blank,Mock,ov-PRDX2)

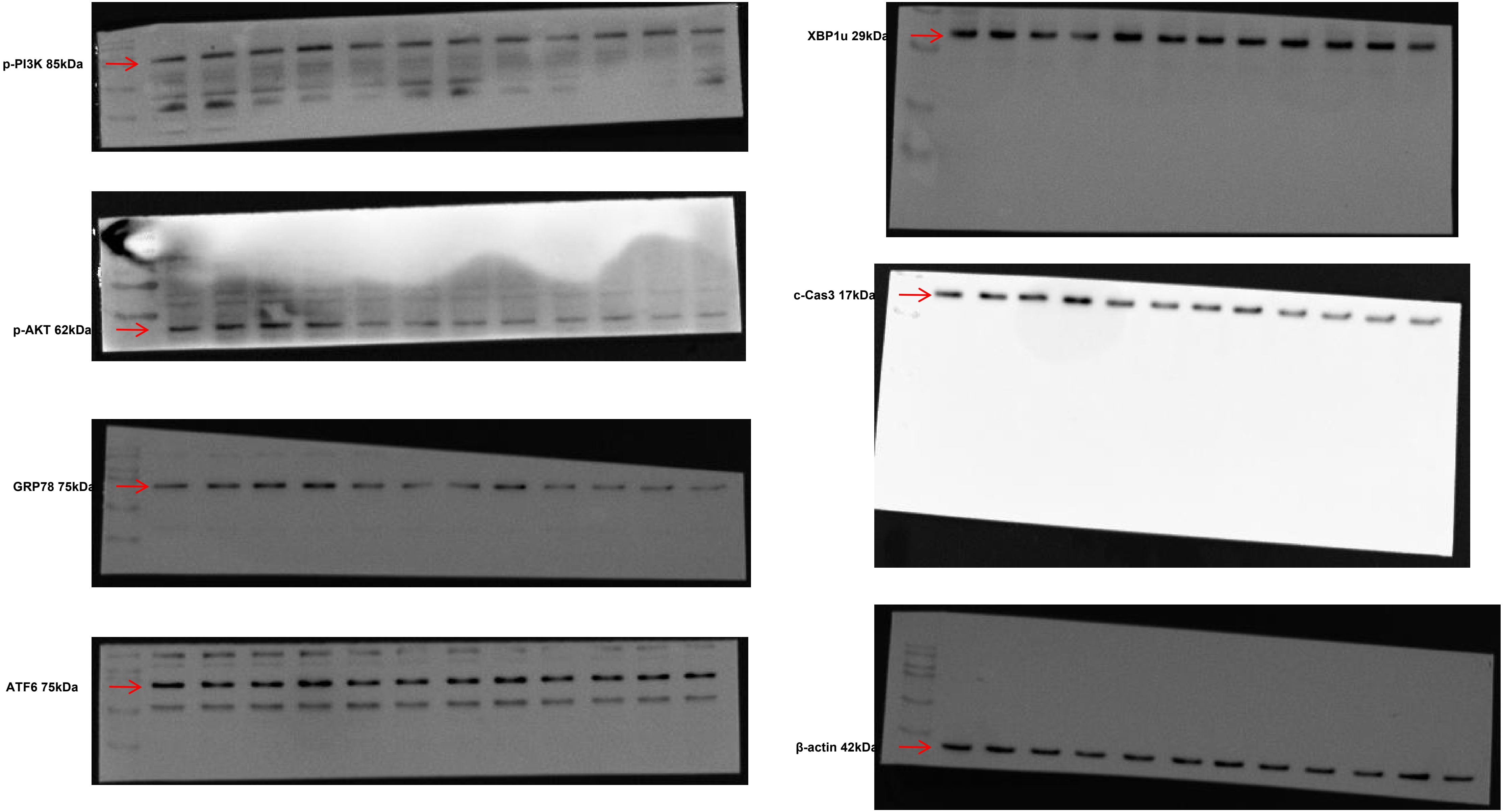

Fig4. C

SiHa Mock(BLM0,12,24,48(h))SiHa ov-PRDX1(BLM0,12,24,48(h))SiHa ov-PRDX2(BLM0,12,24,48(h))

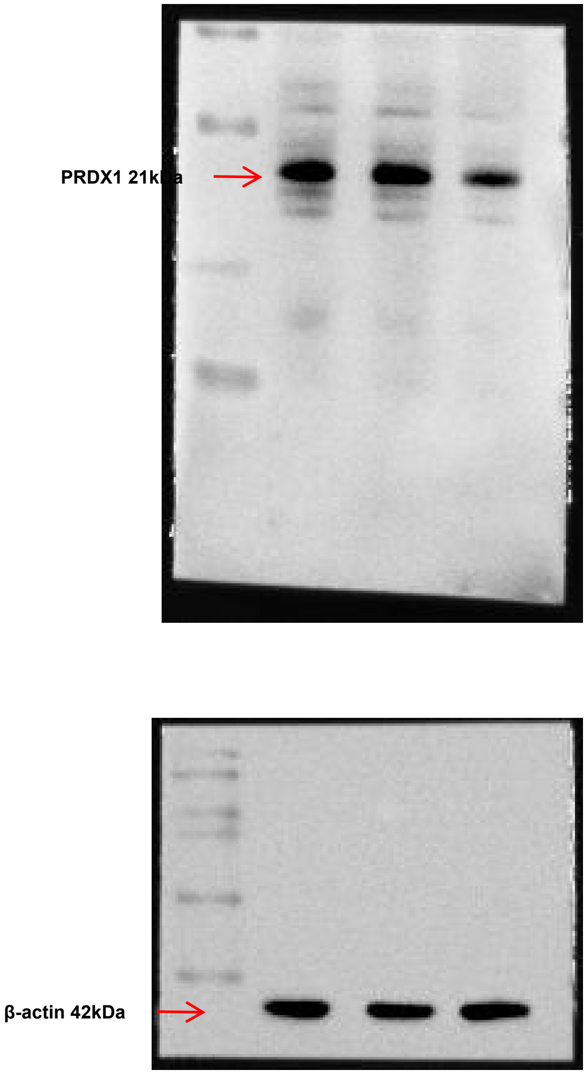

Fig5. A

SiHa (Blank,Mock,sh-PRDX1)

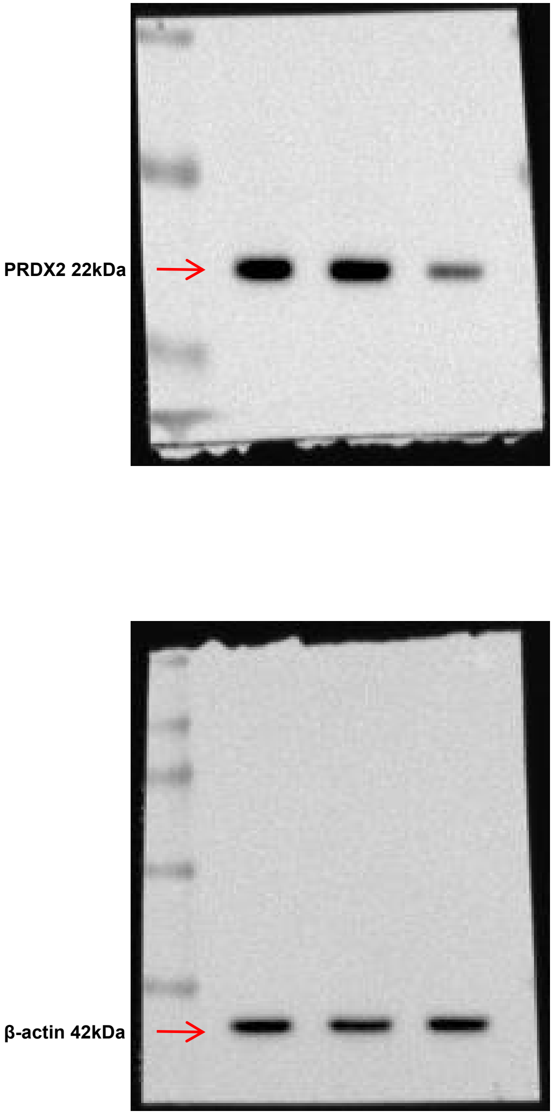

Fig5. B

SiHa (Blank,Mock,sh-PRDX2)

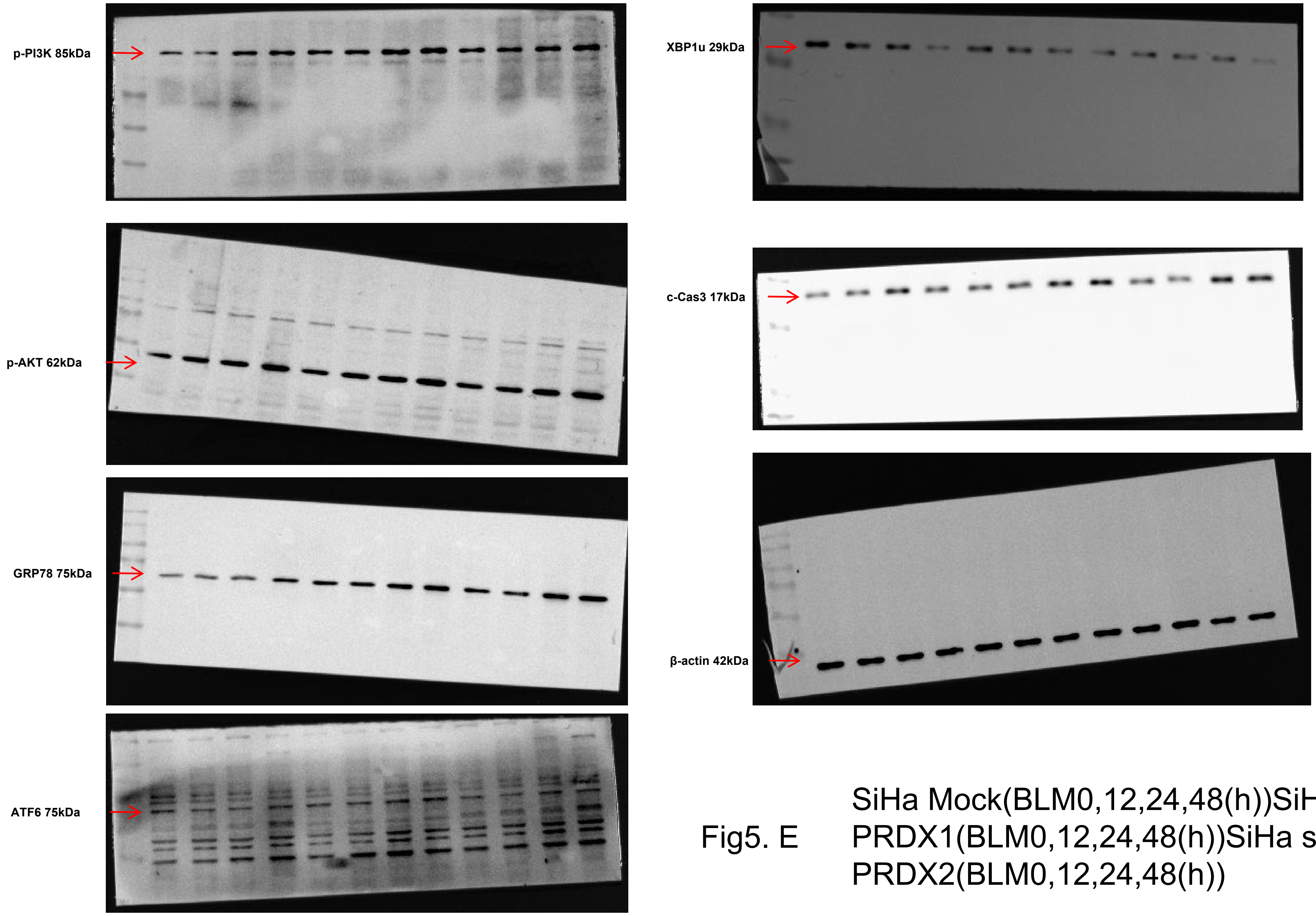

Fig5. E SiHa Mock(BLM0,12,24,48(h))SiHa sh-PRDX1(BLM0,12,24,48(h))SiHa sh-PRDX2(BLM0,12,24,48(h))
